# Conformational study of the protegrin-1 (PG-1) dimer interaction with lipid bilayers and its effect

**DOI:** 10.1186/1472-6807-7-21

**Published:** 2007-04-02

**Authors:** Hyunbum Jang, Buyong Ma, Ruth Nussinov

**Affiliations:** 1 Center for Cancer Research Nanobiology Program, SAIC-Frederick, Inc., NCI-Frederick, Frederick, Maryland 21702, USA; 2 Sackler Inst. of Molecular Medicine, Department of Human Genetics and Molecular Medicine, Sackler School of Medicine, Tel Aviv University, Tel Aviv 69978, Israel

## Abstract

**Background:**

Protegrin-1 (PG-1) is known as a potent antibiotic peptide; it prevents infection via an attack on the membrane surface of invading microorganisms. In the membrane, the peptide forms a pore/channel through oligomerization of multiple subunits. Recent experimental and computational studies have increasingly unraveled the molecular-level mechanisms underlying the interactions of the PG-1 β-sheet motifs with the membrane. The PG-1 dimer is important for the formation of oligomers, ordered aggregates, and for membrane damaging effects. Yet, experimentally, different dimeric behavior has been observed depending on the environment: antiparallel in the micelle environment, and parallel in the POPC bilayer. The experimental structure of the PG-1 dimer is currently unavailable.

**Results:**

Although the β-sheet structures of the PG-1 dimer are less stable in the bulk water environment, the dimer interface is retained by two intermolecular hydrogen bonds. The formation of the dimer in the water environment implies that the pathway of the dimer invasion into the membrane can originate from the bulk region. In the initial contact with the membrane, both the antiparallel and parallel β-sheet conformations of the PG-1 dimer are well preserved at the amphipathic interface of the lipid bilayer. These β-sheet structures illustrate the conformations of PG-1 dimer in the early stage of the membrane attack. Here we observed that the activity of PG-1 β-sheets on the bilayer surface is strongly correlated with the dimer conformation. Our long-term goal is to provide a detailed mechanism of the membrane-disrupting effects by PG-1 β-sheets which are able to attack the membrane and eventually assemble into the ordered aggregates.

**Conclusion:**

In order to understand the dimeric effects leading to membrane damage, extensive molecular dynamics (MD) simulations were performed for the β-sheets of the PG-1 dimer in explicit water, salt, and lipid bilayers composed of POPC lipids. Here, we studied PG-1 dimers when organized into a β-sheet motif with antiparallel and parallel β-sheet arrangements in an NCCN packing mode. We focus on the conformations of PG-1 dimers in the lipid bilayer, and on the correlation between the conformations and the membrane disruption effects by PG-1 dimers. We investigate equilibrium structures of the PG-1 dimers in different environments in the early stage of the dimer invasion. The dimer interface of the antiparallel β-sheets is more stable than the parallel β-sheets, similar to the experimental observation in micelle environments. However, we only observe membrane disruption effects by the parallel β-sheets of the PG-1 dimer. This indicates that the parallel β-sheets interact with the lipids with the β-sheet plane lying obliquely to the bilayer surface, increasing the surface pressure in the initial insertion into the lipid bilayer. Recent experimental observation verified that parallel PG-1 dimer is biologically more active to insert into the POPC lipid bilayer.

## Background

Protegrin-1 (PG-1) is composed of 18 amino-acids (RGGRL-CYCRR-RFCVC-VGR) with a high content of cysteine (Cys) and positively charged arginine (Arg) residues. The peptide displays an antimicrobial activity [1], and thus is a very potent antibiotic peptide [2]. A nuclear magnetic resonance (NMR) study has determined the monomeric structure of PG-1 in solution. The peptide forms a β-hairpin conformation and is stabilized by two disulfide bonds between the cysteine residues [3]. Formation of the two disulfide bonds is crucial for the biological activity of PG-1, since the activity can be restored by stabilizing the peptide structure [4], and the ability to create pores in the membrane depends on its secondary structure [5]. As a cytolytic peptide, PG-1 shares common features with other antimicrobial peptides. These include (i) membrane disruption by forming a pore/channel that leads to cell death [6,7], and (ii) the cationic nature of the peptide which adapts to the amphipathic characteristics [8-10]. However, PG-1 is distinguished from other antimicrobial peptides in that it adopts a β-sheet motif [3], while most antimicrobial peptides have an α-helical structure [11-14].

The major role of PG-1 in the microorganism is the formation of an oligomeric structure in the membrane that creates a pore/channel. It has been suggested that the self-association of PG-1 into a dimeric β-sheet can take place in two ways: an antiparallel β-sheet with a turn-next-to-tail association or a parallel β-sheet with a turn-next-to-turn association [3]. These models initially failed to account for the stable β-sheet conformation of the PG-1 dimer, since instability due to the large electrostatic repulsion between the positively charged arginine residues that cluster at the edge of the dimer interface was encountered. However, the β-sheet dimers can be stabilized when they are associated with lipids, since the interaction of the arginine side chain with the polar lipid head can screen the electrostatic repulsion between neighboring arginine residues. A high-resolution proton NMR study has demonstrated that PG-1 forms a dimeric β-sheet structure in the presence of dodecylphosphocholine (DPC) micelles [15]. The turn-next-to-tail association of PG-1 gives rise to an antiparallel β-sheet, in which six intermolecular backbone hydrogen bonds (H-bonds) are formed between the C-terminal β-strands from each monomer. The β-sheet structure is stable, since the interaction of the arginine side chain with DPC micelle dismisses the electrostatic repulsion.

Oligomerization or aggregation of PG-1 dimers into ordered aggregates is responsible for the membrane disrupting effects. It has been reported that PG-1 is immobilized in palmitoyl-oleyl-phosphatidylcholine (POPC) lipid bilayers, suggesting aggregation in the lipid bilayer [16]. The results from NMR experiments using ^19^F spin diffusion showed that PG-1 dimers are populated in the POPC bilayer, while the dimer fraction is reduced as the peptide concentration decreases [17]. The model of Tang *et al*. for the PG-1 aggregation from 2D solid-state NMR experiments on PG-1 aggregates in phosphate buffer saline (PBS) solution suggested that a parallel β-sheet in an NCCN (where N and C represent N- and C-termini, respectively) packing mode is the sole repeat motif in the ordered aggregates [18]. The recent rotational-echo double-resonance solid-state NMR experiments determined that the PG-1 dimer structure is a β-sheet in the NCCN organization bound to the POPC bilayer [19]. In the presence of anionic lipid in the bilayers, PG-1 is well inserted and begins to aggregate to form an annular pore, while PG-1 aggregates into β-sheets on the surface of POPC/cholesterol biayers [20].

The formation of the PG-1 dimer is important for pore formation in the lipid bilayer, since the dimer can be regarded as the primary unit for assembly into the ordered aggregates. Experimental observations for other cytolytic peptides have verified that dimer seeding accelerates the formation of ordered aggregates, such as fibrils made by β-amyloids and prions [21]. However, in order to understand the molecular mechanism for pore formation and the biological activity of PG-1, one still needs to elucidate a number of crucial points: (i) how do the PG-1 monomers interact to form a β-sheet dimer; (ii) where is the β-sheet of the PG-1 dimer created; and (iii) how does the β-sheet of the PG-1 dimer interact with the lipid bilayer on the molecular level. Computer simulations of detailed atomic models can be a powerful approach to the understanding of the complex protein/lipid system and provide information complementary to experimental approaches. In this paper, we performed extensive molecular dynamics (MD) simulations of the β-sheet of PG-1 dimer in explicit water, salt, and lipid bilayers composed of POPC lipids. Our long term goal is to provide a detailed mechanism of the membrane disrupting effects by cationic peptides with β-sheet structure. The PG-1 dimer can have different β-sheet arrangements in the NCCN packing mode. The conformations are stable at the amphipathic interface of the lipid bilayer, which is consistent with the experimental suggestions [3,15]. We observe membrane disruption effects by the β-sheet, depending on the β-sheet arrangement.

## Results

### Arrangements of β-sheet Conformations

**Figure 1:**
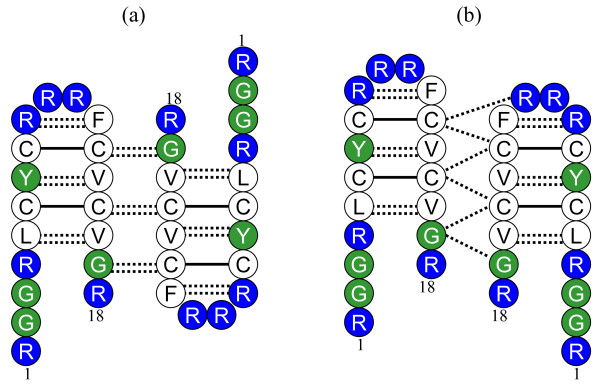
**PG-1 β-sheet arrangements**. Topological diagrams for the PG-1 dimers in the (a) antiparallel and (b) parallel β-sheet arrangements. Both dimers are in an NCCN packing mode. Blue and white beads represent the positively charged (Arg) and hydrophobic residues, respectively, and the polar residue (Tyr) and Gly residues are denoted by green beads. Solid lines indicate the disulfide bonds between Cys residues, and dotted lines indicate the backbone hydrogen bond (H-bond). The first and the last residues for each monomer are indicated by the residue number.

PG-1 dimers in a β-sheet motif were used in the all-atom simulations. In our simulations, PG-1 dimers in two different β-sheet arrangements, antiparallel (turn-next-to-tail) and parallel (turn-next-to-turn) β-sheets in an NCCN packing mode, were considered [18]. Fig. 1 shows topological diagrams for the PG-1 dimers in the (a) antiparallel and (b) parallel β-sheet arrangements. In the NCCN packing mode, the intermolecular interface for both dimers is located in-between the C-terminal strands of the planar β-sheet. Since the experimental crystal structure of the PG-1 dimer is currently unavailable, the simulation used the β-sheets of the PG-1 dimer that were initially assembled from the β-hairpins with two different conformational origins: (i) the β-hairpin resolved by NMR spectroscopy [3] (A1 & P1 β-sheets; A stands for antiparallel, P for parallel), and (ii) the pre-relaxed β-hairpin in the lipid bilayer environment [22] (A2 & P2 β-sheets). Although the topological diagram suggests that there are six possible intramolecular backbone hydrogen bonds (H-bond) between the β-strands within each β-hairpin monomer and also six possible intermolecular backbone H-bonds between the β-hairpin monomers [15,18], in our simulations the initially assembled PG-1 dimers have a smaller number of the backbone H-bonds than in the topology diagram. For the A1 and P1 β-sheets, five intramolecular and four intermolecular backbone H-bonds were initially observed. However, for the A2 and P2 β-sheets, five intramolecular and two intermolecular backbone H-bonds were initially counted. The smaller number of the intermolecular backbone H-bonds in the A2 and P2 β-sheets are the outcome of the cross β-stranded structure of the pre-relaxed β-hairpin in the lipid bilayer [22], preventing maximal contracts of the intermolecular backbone H-bonds between the C-terminal strands from each monomer. Although, in our simulations the A2 and P2 β-sheets have fewer intermolecular backbone H-bonds than the A1 and P1 β-sheets, the dimer interface for the A2 and P2 β-sheets is held tightly by two intermolecular backbone H-bonds between two cysteine residues. These residues have lower fluctuations due to the disulfide bond. The different numbers of intermolecular backbone H-bonds observed for the different β-sheets reflect the fact that the β-sheet dimer can be at different locations, e.g. dimer formation in bulk or on/in the membrane.

### Dimeric β-sheet conformations at different environments

**Figure 2:**
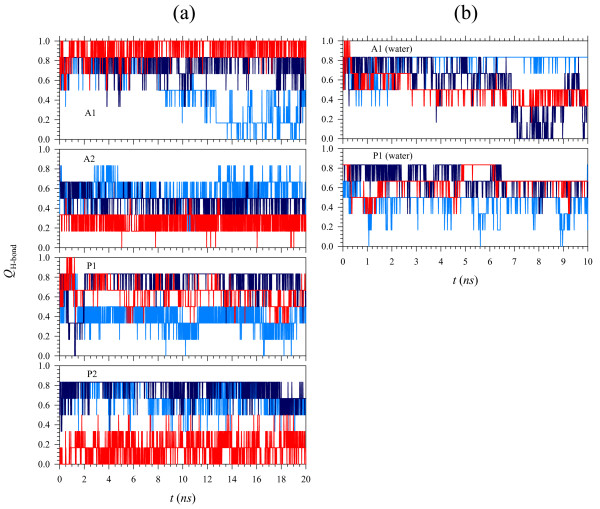
**Backbone hydrogen bond**. Fractions of intermolecular and intramolecular backbone hydrogen bonds (H-bonds), *Q*_H-bond_, as a function of the time, *t*, (a) for the antiparallel (A1 & A2 – 1 st & 2 nd rows) and parallel (P1 & P2 – 3 rd & 4th rows) β-sheets at the amphipathic interface of the lipid bilayer, and (b) for the antiparallel (A1) and parallel (P1) β-sheets in water. The red lines denote the fraction of intermolecular H-bond at the dimer interface, and the light and dark blue lines denote the fractions of intramolecular H-bond within each β-hairpin monomer.

Figure 2(a) illustrates the time-dependent fractions of intermolecular (red lines) and intramolecular (light and dark blue lines for each β-hairpin monomer) backbone hydrogen bonds (H-bonds), *Q*_H-bond_, for the antiparallel (A1 & A2) and parallel (P1 & P2) β-sheets of PG-1 dimer at the amphipathic interface of the lipid bilayer. We calculated the fraction using *Q*_H-bond _= *N*_H-bond_/ NH-bondmax⁡
 MathType@MTEF@5@5@+=feaafiart1ev1aaatCvAUfKttLearuWrP9MDH5MBPbIqV92AaeXatLxBI9gBaebbnrfifHhDYfgasaacH8akY=wiFfYdH8Gipec8Eeeu0xXdbba9frFj0=OqFfea0dXdd9vqai=hGuQ8kuc9pgc9s8qqaq=dirpe0xb9q8qiLsFr0=vr0=vr0dc8meaabaqaciaacaGaaeqabaqabeGadaaakeaacqWGobGtdaqhaaWcbaGaeeisaGKaeeyla0IaeeOyaiMaee4Ba8MaeeOBa4MaeeizaqgabaGagiyBa0MaeiyyaeMaeiiEaGhaaaaa@397E@, where *N*_H-bond _is the number of the intermolecular and intramolecular backbone H-bonds that are monitored during the simulations, and NH-bondmax⁡
 MathType@MTEF@5@5@+=feaafiart1ev1aaatCvAUfKttLearuWrP9MDH5MBPbIqV92AaeXatLxBI9gBaebbnrfifHhDYfgasaacH8akY=wiFfYdH8Gipec8Eeeu0xXdbba9frFj0=OqFfea0dXdd9vqai=hGuQ8kuc9pgc9s8qqaq=dirpe0xb9q8qiLsFr0=vr0=vr0dc8meaabaqaciaacaGaaeqabaqabeGadaaakeaacqWGobGtdaqhaaWcbaGaeeisaGKaeeyla0IaeeOyaiMaee4Ba8MaeeOBa4MaeeizaqgabaGagiyBa0MaeiyyaeMaeiiEaGhaaaaa@397E@ is the maximum possible number of the backbone H-bonds as described in the topological diagram of Fig. 1. It can be seen from the figure that the dimer interface in the antiparallel β-sheets (A1 & A2) is more stable than that in the parallel β-sheets (P1 & P2), since for the antiparallel β-sheets, the fluctuations of the red lines that represent the formation of the intermolecular backbone H-bonds are less than those corresponding to the parallel β-sheets. However, the large fluctuations in the formation of the intermolecular backbone H-bonds observed in the parallel β-sheets are closely related to the β-sheet activity on the lipid bilayer. All the β-sheets appear stable with the backbone H-bonds fluctuating near initial values.

In the bulk water environment, the β-sheets structures of PG-1 dimer are less stable, since in Fig. 2(b) large fluctuations in the *Q*_H-bond _lines are observed. To compare the dimeric behavior between the water and on the lipid, the β-sheets used in the water simulation are the same (A1 and P1) as those used in the lipid simulation. Although the PG-1 dimers in water exhibit a partially ordered or slightly collapsed β-sheet conformation compared to the β-sheet conformations on the lipid bilayer, two intermolecular backbone H-bonds between two cysteine residues at the C-terminal strands from each monomer retain strongly the dimer interface. The formation of PG-1 dimer in water implies that the PG-1 dimer can exist in many different environments and acts as a seed in the formation of ordered aggregates in a proper environment.

**Figure 3:**
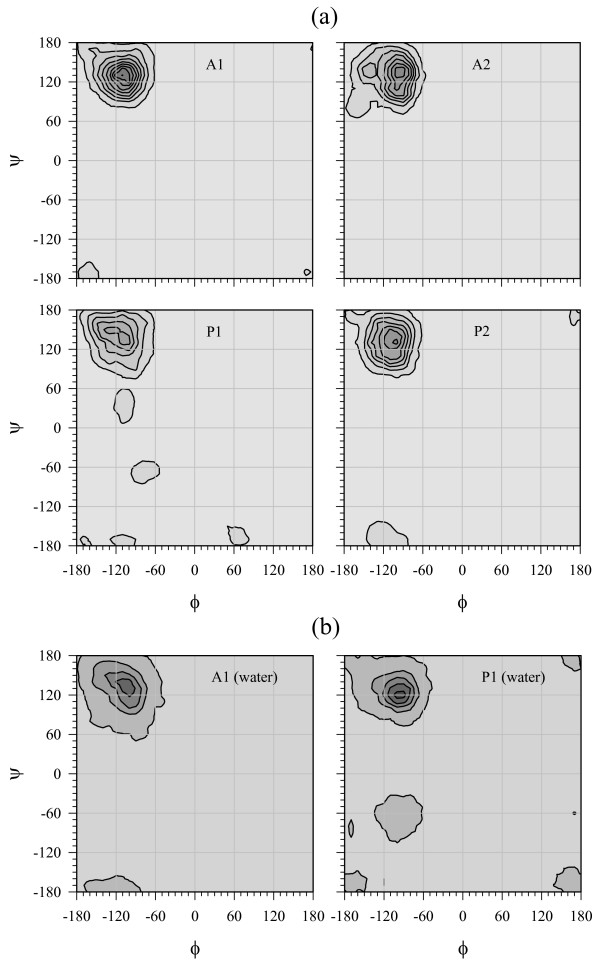
**Backbone dihedral angle**. Backbone dihedral angle distributions (a) for the antiparallel (A1 & A2) and parallel (P1 & P2) β-sheets at the amphipathic interface of the lipid bilayer, and (b) for the antiparallel (A1) and parallel (P1) β-sheets in water. Dihedral angles (φ, ψ) are calculated only for the residues in the β-strands (residues 4 to 8 and 13 to 17 of each β-hairpin).

The detailed secondary structures for the β-sheet conformations are investigated. Fig. 3 shows the contour maps, representing the distributions of backbone dihedral angles of φ and ψ for the residues in the β-strands. In Fig. 3(a) the contour maps for the β-sheets on the lipid bilayer suggest that the PG-1 dimers preserve the β-sheet structure during the simulation, since the contour lines encompass a high population at the region that represents the β-sheet characteristics. In Fig. 3(b) the contour maps for the β-sheets in water suggest that the PG-1 dimers also preserve the β-sheet structure during the simulation. But the wide distributions of φ and ψ angles suggest that the β-sheets in water are flexible, leading to a slightly collapsed or partially folded β-sheet conformation.

**Figure 4:**
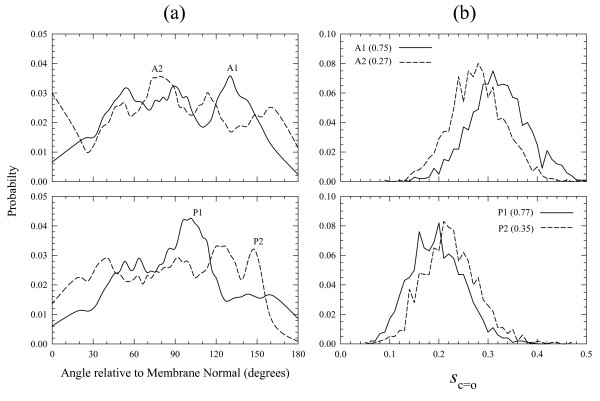
**Peptide order parameter**. (a) The probability distribution of the angle between the backbone carbonyl bond, C=O, in the β-strands and the normal to the lipid bilayer surface for the antiparallel (A1 (solid line) & A2 (dotted line)) and parallel (P1 (solid line) & P2 (dotted line)) β-sheets. (b) The probability distribution of the peptide order parameter, *S*_C=O_, for the antiparallel (A1 (solid line) & A2 (dotted line)) and parallel (P1 (solid line) & P2 (dotted line)) β-sheets. The numbers in the bracket denote the initial values of *S*_C=O _in the starting points.

The orientation of the PG-1 β-sheet on the lipid bilayer is monitored by calculating the angle between the backbone carbonyl bond, C=O, and the normal to the bilayer surface. Only the backbone carbonyl bonds located in residues that form the β-strands (residues 4 to 8 and 13 to 17 of each β-hairpin) are considered in the calculation. The probability distribution for the angle of the C=O vector relative to the membrane normal is shown in Fig. 4(a). At the starting point, the PG-1 β-sheet is in contact with the lipid bilayer, with the β-sheet plane parallel to the bilayer surface. For a perfect planar β-sheet, the distribution curve might be located at a right angle, since all C=O vectors should lie on the membrane surface. However, the peaks in the distribution curve located at many places indicate that the PG-1 β-sheets are not perfectly planar. That is, the β-sheet plane is slightly bent or lies with an oblique angle to the bilayer surface.

For an α-helical peptide, the peptide orientation in the lipid bilayer can be measured experimentally by polarized ATR-FTIR spectroscopy [23,24], and computationally by a calculation of the peptide order parameter [25,26]. We calculate the order parameter for the PG-1 β-sheets on the lipid bilayer, with the same method as that used in the recent simulations [25,26]. The peptide order parameter can be defined by

SC=O=1N∑k=1N(3cos⁡2θij−12),
 MathType@MTEF@5@5@+=feaafiart1ev1aaatCvAUfKttLearuWrP9MDH5MBPbIqV92AaeXatLxBI9gBaebbnrfifHhDYfgasaacH8akY=wiFfYdH8Gipec8Eeeu0xXdbba9frFj0=OqFfea0dXdd9vqai=hGuQ8kuc9pgc9s8qqaq=dirpe0xb9q8qiLsFr0=vr0=vr0dc8meaabaqaciaacaGaaeqabaqabeGadaaakeaacqWGtbWudaWgaaWcbaGaee4qamKaeeypa0Jaee4ta8eabeaakiabg2da9maalaaabaGaeGymaedabaGaemOta4eaamaaqahabaWaaeWaaeaadaWcaaqaaiabiodaZiGbcogaJjabc+gaVjabcohaZnaaCaaaleqabaGaeGOmaidaaOGaeqiUde3aaSbaaSqaaiabdMgaPjabdQgaQbqabaGccqGHsislcqaIXaqmaeaacqaIYaGmaaaacaGLOaGaayzkaaaaleaacqWGRbWAcqGH9aqpcqaIXaqmaeaacqWGobGta0GaeyyeIuoakiabcYcaSaaa@4B62@

where θ_*ij *_is the angle between two vectors of the backbone carbonyl bonds, C=O, in the *i *and *j *residues, and *N *is the total number of the vector pairs. In the calculation, only the C=O bonds located in β-strands (residues 4 to 8 and 13 to 17 of each β-hairpin) are considered. Fig. 4(b) shows the probability distribution of the peptide order parameter, *S*_C=O_, over the 20 *ns *trajectories. For the A1 and P1 β-sheets, where the initial β-hairpin monomer structure is from NMR [3], the initial values of *S*_C=O _are 0.75 and 0.77, respectively. These values are much smaller for the A2 and P2 β-sheets, where the initial β-hairpin monomer structure is from our previous simulation [22], which are 0.27 and 0.35, respectively. However, these initial values of the peptide order parameter are not preserved for the β-sheets with the same monomer origin during the simulations. The distributions of *S*_C=O _reveal that for the antiparallel β-sheets (A1 & A2), the distribution curves are located around *S*_C=O _= 0.3, while the curves are found around *S*_C=O _= 0.2 for the parallel β-sheets (P1 & P2). Both parallel β-sheets have smaller values of the peptide order parameter, indicating that the values of the peptide order parameter strongly depend on the β-sheet topology.

### Interactions of PG-1 β-sheets with Lipids

**Figure 5:**
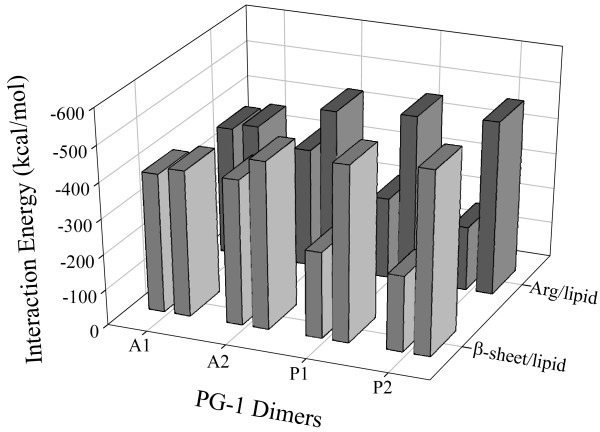
**Lipid interaction energy**. Interaction energy of the PG-1 β-sheets with lipids (light gray bars), and interaction energy of the Arg residues with lipids (dark gray bars). The interaction energy is calculated separately for each monomer in the PG-1 β-sheet.

The interaction energy between the PG-1 β-sheets and lipids is calculated in order to understand the dominant forces that stabilize the β-sheet structure. Fig. 5 shows the averaged interaction energies of the PG-1 β-sheets with the lipid (light gray bars) for the different β-sheet conformations. The figure also presents the averaged interaction energies between the Arg residues and lipid (dark gray bars). The interaction energy is calculated every 10 *ps *and averaged over the 20 *ns *simulations for each monomer separately. For the antiparallel β-sheets (A1 & A2), the strength of the interaction energy of each monomer with the lipid is similar. However, for the parallel β-sheets (P1 & P2), at least one monomer interacts with the lipid more strongly than the other. The strong interaction reflects the strong electrostatic interaction of the Arg residues with the lipid headgroups. For the P1 β-sheet, the terminal Arg residues of the monomer whose apolar surface faces the bilayer surface closely contact the lipid, while for the P2 β-sheet the loop Arg residues of the monomer whose apolar surface faces the bulk region interact strongly with lipid. These strong Arg-lipid interactions observed in the parallel PG-1 β-sheet are closely related to the bending or tilting of the β-sheet, ultimately causing the membrane thinning and disruption.

**Figure 6:**
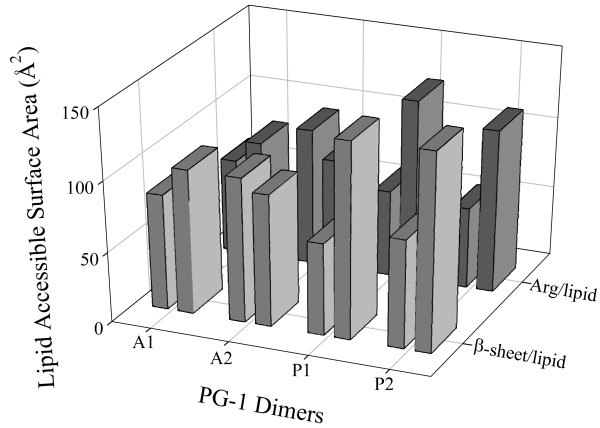
**Lipid accessible surface area**. Lipid accessible surface areas for the PG-1 β-sheets (light gray bars) and the Arg residues (dark gray bars). The lipid accessible surface area is calculated separately for each monomer in the PG-1 β-sheet.

Fig. 6 shows the averaged lipid accessible surface area for the PG-1 β-sheets in different arrangements (light gray bars). The lipid accessible surface area is calculated every 10 *ps *and averaged over the 20 *ns *simulations for each monomer separately. The figure also presents the averaged lipid accessible surface areas for the Arg residues (dark gray bars). As expected, the lipid accessible surface area is strongly correlated with the interaction energy of the PG-1 β-sheets as seen in Fig. 5. Larger lipid accessible surface area yields larger interaction energy. This is also true for the Arg residues, especially for the parallel PG-1 β-sheets, suggesting that the Arg side chains in parallel β-sheets are indeed located at the deep amphipathic interface, perturbing the polar lipid heads in the lipid bilayer.

### Membrane Disruption Effects by PG-1 β-sheets

**Figure 7:**
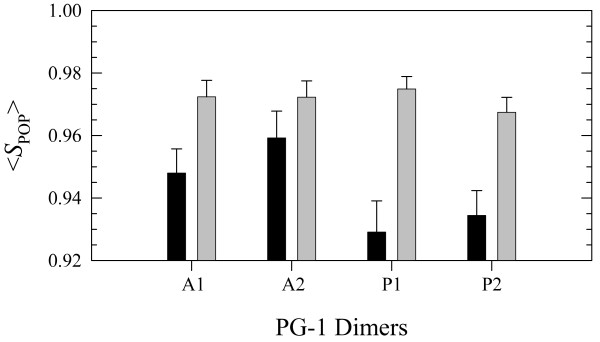
**Plane order parameter**. Plane order parameter of the bilayer surface, *S*_POP_, at the top leaflet (solid bars) and at the bottom leaflet (gray bars) of the lipid bilayer. The PG-1 β-sheet is located at the top leaflet of the lipid bilayer.

In our previous study of the PG-1 monomer on the lipid bilayers [22], we have shown that the PG-1 β-hairpin indeed induced the thinning effect in the lipid bilayer containing anionic lipids, while no thinning effect was observed for the pure lipid bilayer composed of POPC. To observe a similar effect induced by the PG-1 dimer, the degree of flatness or roughness of the bilayer surface plane is monitored during the simulations. We introduce the plane order parameter of the bilayer surface, *S*_POP_, which calculates the angle between the positional vector connecting two adjacent phosphate atoms and the plane of the bilayer surface,

SPOP=∑<i,j>(1−(zjP−ziP)2rij2)12,
 MathType@MTEF@5@5@+=feaafiart1ev1aaatCvAUfKttLearuWrP9MDH5MBPbIqV92AaeXatLxBI9gBaebbnrfifHhDYfgasaacH8akY=wiFfYdH8Gipec8Eeeu0xXdbba9frFj0=OqFfea0dXdd9vqai=hGuQ8kuc9pgc9s8qqaq=dirpe0xb9q8qiLsFr0=vr0=vr0dc8meaabaqaciaacaGaaeqabaqabeGadaaakeaacqWGtbWudaWgaaWcbaGaeeiuaaLaee4ta8Kaeeiuaafabeaakiabg2da9maaqafabaWaaeWaaeaacqaIXaqmcqGHsisldaWcaaqaaiabcIcaOiabdQha6naaDaaaleaacqWGQbGAaeaacqqGqbauaaGccqGHsislcqWG6bGEdaqhaaWcbaGaemyAaKgabaGaeeiuaafaaOGaeiykaKYaaWbaaSqabeaacqaIYaGmaaaakeaacqWGYbGCdaqhaaWcbaGaemyAaKMaemOAaOgabaGaeGOmaidaaaaaaOGaayjkaiaawMcaaaWcbaGaeyipaWJaemyAaKMaeiilaWIaemOAaOMaeyOpa4dabeqdcqGHris5aOWaaWbaaSqabeaadaWcaaqaaiabigdaXaqaaiabikdaYaaaaaGccqGGSaalaaa@525E@

where *r*_ij _is the distance between two phosphate atoms, and ziP
 MathType@MTEF@5@5@+=feaafiart1ev1aaatCvAUfKttLearuWrP9MDH5MBPbIqV92AaeXatLxBI9gBaebbnrfifHhDYfgasaacH8akY=wiFfYdH8Gipec8Eeeu0xXdbba9frFj0=OqFfea0dXdd9vqai=hGuQ8kuc9pgc9s8qqaq=dirpe0xb9q8qiLsFr0=vr0=vr0dc8meaabaqaciaacaGaaeqabaqabeGadaaakeaacqWG6bGEdaqhaaWcbaGaemyAaKgabaGaeeiuaafaaaaa@30D8@ and zjP
 MathType@MTEF@5@5@+=feaafiart1ev1aaatCvAUfKttLearuWrP9MDH5MBPbIqV92AaeXatLxBI9gBaebbnrfifHhDYfgasaacH8akY=wiFfYdH8Gipec8Eeeu0xXdbba9frFj0=OqFfea0dXdd9vqai=hGuQ8kuc9pgc9s8qqaq=dirpe0xb9q8qiLsFr0=vr0=vr0dc8meaabaqaciaacaGaaeqabaqabeGadaaakeaacqWG6bGEdaqhaaWcbaGaemOAaOgabaGaeeiuaafaaaaa@30DA@ are the height of the phosphate atoms along the bilayer normal at the *i*th and *j*th residue, respectively. The notation <*i*,*j*> means that the sum is restricted to adjacent pairs of phosphate atoms. The plane order parameter *S*_POP _can measure the flatness or roughness of the bilayer surface, indicating that for *S*_POP _< 1 the bilayer surface is a perfect smooth plane, while for *S*_POP _< 1 the bilayer surface is bent or contains many troughs. Fig. 7 shows the averaged plane order parameter, <*S*_POP _>, at the top leaflet (solid bars) and at the bottom leaflet (gray bars) of the lipid bilayer during the simulations. The PG-1 β-sheet is located at the top leaflet of the lipid bilayer. As expected, the bilayer surface at the top leaflet is rougher than the bottom leaflet of the lipid bilayer, since the top leaflet contains the β-sheet. Note however that the bilayer surface at the top leaflet containing the parallel β-sheets (P1 & P2) is rougher than that at the same leaflet containing the antiparallel β-sheets (A1 & A2). This suggests that the parallel β-sheets are very active and more strongly interact with the lipid bilayer, with the activity closely related to the bilayer disruption.

**Figure 8:**
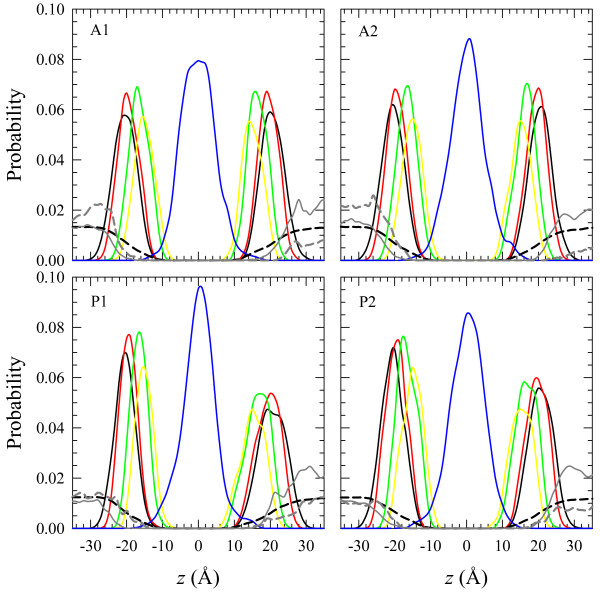
**Solvent distribution**. Probability distribution functions (*P*) for different component groups of lipid (*P*_Chol _(choline – black lines), *P*_PO4 _(phosphate – red lines), *P*_Glyc _(glycerol – green lines), *P*_Carb _(carbonyls – yellow lines), and *P*_CH3 _(methyl – blue lines)), and for other solvents (*P*_water _(water – dashed black lines), *P*_Na _(sodium ion – dashed gray lines), and *P*_Cl _(chloride ion – gray lines)) as a function of distance from the bilayer center for different PG-1 dimers on the lipid bilayer composed of POPC.

The disruption of the lipid bilayer reflects disordering of lipid molecules around the PG-1 β-sheets. The average positions of lipid groups may illustrate how lipids are distributed in the bilayer in response to the dimer invasion. Fig. 8 shows the position probability distribution functions (*P*) for five different component groups of POPC, water, and salts as a function of the distance from the POPC lipid bilayer center. The POPC headgroup is divided into four subunits, choline (*P*_Chol_, black lines), phosphate (*P*_PO4_, red lines), glycerol (*P*_Glyc_, green lines), and carbonyl (*P*_Carb_, yellow lines). The tail group involves two fatty acids with terminal methyl (*P*_CH3_, blue lines). The PG-1 β-sheet is located at the top leaflet of the lipid bilayer (positive *z *area), while the bottom leaflet of the lipid bilayer (negative *z *area) contains lipids only. For the antiparallel β-sheets (A1 & A2), the symmetric distributions of the lipid headgroups at both sides of the bilayer indicate that there are no disturbances in the lipid arrangement induced by the β-sheets. However, for the parallel β-sheets (P1 & P2), the asymmetric distributions of the lipid headgroups indicate that there are great disturbances in the lipid arrangement, especially at the top leaflet of the lipid bilayer. This is consistent with the result presented in Fig. 7 that the bilayer surface containing the parallel β-sheet is very rough, resulting from the disordered lipid headgroups. This leads to the bilayer disruption. In the distributions of salts, the probability for finding chloride ions is very high near the bilayer surface at the top leaflet, since the PG-1 β-sheet contains many positive charges.

To investigate the average structure in the interior of the bilayer, the deuterium order parameter, *S*_CD_, was calculated using

**Figure 9:**
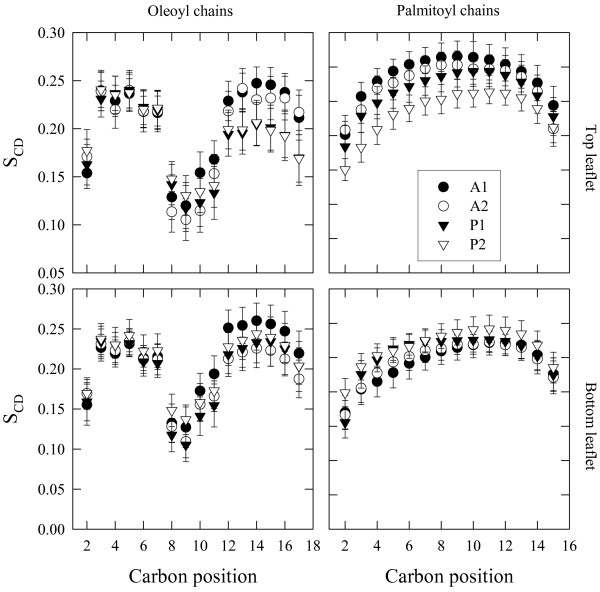
**Lipid order parameter**. Deuterium order parameters, S_CD_, for the oleoyl (left column) and palmitoyl (right) chains of the lipids at the top leaflet (peptide-containing) (upper panel) and the bottom leaflet (lower) of the lipid bilayer composed of POPC for the antiparallel β-sheets (A1 (solid circles) & A2 (open circles)) and the parallel β-sheets (P1 (solid triangles) & P2 (open triangles)).

SCD=12〈3cos⁡2θ−1〉,
 MathType@MTEF@5@5@+=feaafiart1ev1aaatCvAUfKttLearuWrP9MDH5MBPbIqV92AaeXatLxBI9gBaebbnrfifHhDYfgasaacH8akY=wiFfYdH8Gipec8Eeeu0xXdbba9frFj0=OqFfea0dXdd9vqai=hGuQ8kuc9pgc9s8qqaq=dirpe0xb9q8qiLsFr0=vr0=vr0dc8meaabaqaciaacaGaaeqabaqabeGadaaakeaacqWGtbWudaWgaaWcbaGaee4qamKaeeiraqeabeaakiabg2da9maalaaabaGaeGymaedabaGaeGOmaidaamaaamqabaGaeG4mamJagi4yamMaei4Ba8Maei4Cam3aaWbaaSqabeaacqaIYaGmaaGccqaH4oqCcqGHsislcqaIXaqmaiaawMYicaGLQmcacqGGSaalaaa@3FAA@

where θ is the angle between the C-H bond vector and the membrane normal, and the angular brackets indicate averaging over time and over lipids. Fig. 9 shows the order parameters for the oleoyl (left column) and palmitoyl (right) chains for the lipid bilayers composed of POPC for the antiparallel β-sheets (A1 (solid circles) & A2 (open circles)) and the parallel β-sheets (P1 (solid triangles) & P2 (open triangles)). Lipids at the top leaflet (peptide-containing, upper panel) and the bottom leaflet (lower) of the lipid bilayer are considered separately. The bars represent the standard deviation errors. At the top leaflet, the order parameters for the lipids with the antiparallel β-sheets are slightly higher than those for the lipids with the parallel β-sheets. At the bottom leaflet, however, the order parameters for the lipids are similar. Lower order parameters for the lipids containing the parallel β-sheets indicate that the lipids have significantly disordered tails, causing the disruption of the lipid bilayer.

## Discussion

**Figure 10:**
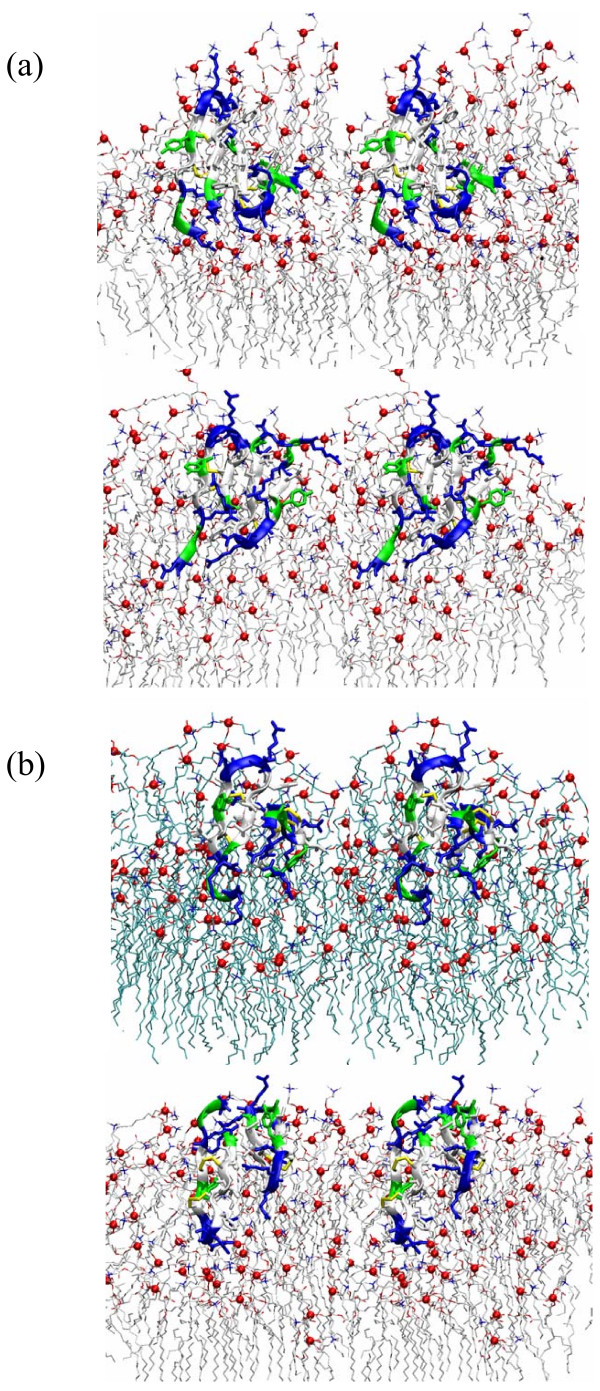
**PG-1 β-sheet conformations**. Cartoons of PG-1 β-sheets in stereo view, representing the final peptide conformation on the lipid bilayer for the antiparallel ((a) A1 and (b) A2) and parallel ((c) P1 and (d) P2) β-sheets. In the cartoons, hydrophobic residues and disulfide bonded Cys residues are shown in white, a polar residue (Tyr) and Gly are shown in green, and positively charged residues (Arg) are shown in blue. Disulfide bonds are highlighted in yellow. For clarity, only the top leaflet of the lipid bilayer is shown, and phosphate atoms are denoted as red beads.

We simulated the Protegrin-1 (PG-1) dimers with different β-sheet arrangements in an aqueous solution and in a lipid bilayer composed of POPC. To create the PG-1 dimer, the two β-hairpins were initially assembled into β-sheets with both antiparallel (turn-next-to-tail) and parallel (turn-next-to-turn) β-sheet motifs in an NCCN packing mode [18]. The monomer β-hairpin conformations were obtained from NMR spectroscopy [3] and our previous PG-1 monomer simulations [22]. Although, the β-sheets of the PG-1 dimer have different monomer origins, the behavior of the β-sheets on the bilayer surface strongly depends on their topology, i.e parallel or antiparallel arrangement which is more important for the lipid interaction. Thus, the energetically stable PG-1 dimer conformation strongly depends on its surrounding environments. While the β-sheet conformations are less stable in the bulk water environment, both the antiparallel and parallel β-sheet conformations of the PG-1 dimer are well preserved at the amphipathic interface of the lipid bilayer, with the dimer interface of the antiparallel β-sheets being more stable. In all cases, the dimer interface is held tightly by at least two intermolecular backbone H-bonds between two cysteine residues at the C-terminal strands from each monomer. As suggested by the experimental observations [3,15], the dimeric β-sheet conformations on the lipid bilayer are stable when the β-sheets are associated with the lipids, since the strong electrostatic repulsion between the β-hairpin monomers can be shaded by the lipids. However, the β-sheet itself has a significant conformational change to avoid the repulsive force due to the neighboring arginine side chains. This leads to a twisted or cross β-sheet structure. Fig. 10 shows snapshots of the β-sheets of the PG-1 dimer at the end of the simulations. It can be seen from the figure that significant twist of the β-sheet conformation, especially at the loop and both termini regions, can be observed for all β-sheets on the bilayer surface.

In this work we wish to compare between different dimer conformations in different environments and to find the connection between the dimer conformation and the activity of the PG-1 dimer in the early stage of the membrane disruption. In this early stage, the PG-1 dimer is in contact with the bilayer surface, preparing the penetration into the hydrophobic core region of the lipid bilayer. Thus, our lipid simulations start with a PG-1 dimer that is initially located at the top of the lipid bilayer without giving any stress to the lipid bilayer. The dimer quickly moves to the amphipathic interface immediately following the start of the simulation. This indicates that our simulation results are independent of the initial dimer location with respect to the interface, since this kind of relaxation takes a very short time. If the PG-1 monomer or dimer is fully embedded in the lipid bilayer, the membrane disruption effects are enhanced. In this case, the initial position and orientation of the peptides in the lipid bilayer are crucial, since the peptides cause hydrophobic mismatch between the lipid bilayer and the PG-1, inducing a highly curved bilayer surface. In this paper, we do not target the dimer insertion into the lipid bilayer, since the timescale for such spontaneous dimer relocation may range from few hundreds of nanoseconds to a microsecond, which is far beyond the timescale of our simulations. In our simulation, the antiparallel PG-1 β-sheets bind within the top leaflet of a lipid bilayer with its apolar surface of the β-sheet containing the four disulfide bonds facing the bilayer surface, closely following the experimental suggestion [3]. Unlike the parallel PG-1 β-sheet with the symmetric β-sheet surfaces, the antiparallel PG-1 β-sheet has the asymmetry of the β-sheet surfaces. The antiparallel PG-1 β-sheet may bind the lipid bilayer with its four disulfide bonds away from the bilayer surface. However, the simulation results for the opposite-facing interaction of the antiparallel β-sheet are not expected to be different from the observations in our current setting, since most charged residues are located at both ends of the β-sheet of the PG-1 dimer and do not alter the charge distribution following the β-sheet flip. Electrostatic interactions between the positively charged residues of the PG-1 dimer and the lipid headgroups play an important role in the peptide/lipid interaction.

The simulations of the PG-1 β-sheets with different monomer β-hairpin conformations have shown that the behavior of the β-sheets on the bilayer surface strongly depends on the topology. For example, parallel β-sheets have smaller values of the peptide order parameter, stronger lipid interaction, and they induce a bilayer disruption effect. In the turn-next-to-turn association of the parallel β-sheet, charge distributions due to the positively charged arginine side chains are separated into two regions, β-turns and both termini. As compared to the flexible motions in the arginine residues in order to avoid electrostatic repulsion at both termini, the dynamic motions of the arginine side chains at the β-turns are stiffened due to the restricted backbone motions, clustering positively charges at the one side of the β-sheet. These confined repulsive forces by the positive charges at the β-turns induce distortion of the β-sheet plane, lying obliquely to the bilayer. As a result, one monomer of the parallel β-sheets interacts with the lipid more strongly than the other and has a larger lipid accessible surface area than the other as seen in Fig. 5 and 6. It seems that in the initial stage of the dimer invasion into the lipid bilayer, the oblique attack by the parallel β-sheets into the lipid bilayer increases the bilayer surface pressure and hence enhances the membrane disruption effects. The aggregation of PG-1 dimers into ordered aggregates accomplishes the membrane disrupting effects via forming a pore/channel in the membrane [20]. Based on our observations, we deduce that the parallel β-sheet is an active candidate to insert into the lipid bilayer and to be the sole repeat motif in the ordered aggregates as suggested by the solid-state NMR experiments [18,19].

In our previous PG-1 monomer simulations [22], PG-1 exhibited different features at the amphipathic interface of the lipid bilayers with different lipid compositions. In the presence of anionic lipids in the bilayer, PG-1 located more closely to the bilayer surface and interacted more strongly with the lipids. The local thinning of the lipid bilayer due to the PG-1 invasion was clearer in the lipid bilayer with anionic lipids. On the other hand, no thinning effect due to the PG-1 monomer was observed in the pure POPC lipid bilayer. In this paper, we do not observe any influence on the POPC lipid bilayer due to the antiparallel β-sheets. However, a slight bending or disruption of the lipid bilayer within the parallel β-sheets can be clearly seen. In fact antimicrobial activity of the peptide would be warranted in lipid bilayers containing anionic lipid. This is also our interest, to use the anionic lipid in the future. To date, many experimental studies have used the POPC bilayer systems [16,17,19]. However, recently, membrane containing anionic lipid has been used to demonstrate the formation of PG-1 pore in the membrane [20].

The model of PG-1 dimerization in the POPC lipid bilayer [17] has suggested that at low peptide concentration the PG-1 monomer likely remains on the bilayer surface and PG-1 dimers are inserted into the lipid bilayer. At high concentration, the dimer fraction increases, and the ordered aggregates form a toroidal pore in the lipid bilayer. However, there was no clear indication where the dimerization of PG-1 takes place to create the β-sheet structure. Our previous simulation showed that the monomer β-hairpin structure of PG-1 is well preserved at the amphipathic interface of the lipid bilayers [22]. Recently, solid-state NMR also showed that the PG-1 monomer is fully immersed in the lipid bilayer [27,28]. Based on these observations, the PG-1 dimerization can occur either on the surface of the lipid bilayer or in the interior of the bilayer, since the PG-1 monomers are populated in both environments. Furthermore, our current simulation showed that the PG-1 dimer in water exhibits a partially folded β-sheet conformation. The formation of the dimer in water implies that the dimerization can occur in many different environments and that as a seed in the formation of ordered aggregates, the PG-1 dimer can directly insert into the lipid bilayer. On the bilayer surface, the β-sheets of PG-1 dimer disturb the lipid order through strong electrostatic interaction between the polar headgroup and the charged side chain of the β-sheet, inducing local thinning, and facilitating dimeric insertion. In the interior of the lipid bilayer, the embedded β-sheet of the PG-1 dimer causes hydrophobic mismatch between the lipid bilayer and the PG-1 dimer, inducing highly curved bilayer surface. In both cases, the PG-1 dimer is responsible for the membrane disruption/thinning effects, irrespective of where the PG-1 β-sheet is located on/in the lipid bilayer. Continued investigations of PG-1 dimers embedded in the lipid bilayer and of ordered aggregates of the PG-1 complex in the setting of the lipid bilayers are being conducted to further our understanding of the complex behavior of membrane disruption.

## Conclusion

Here, we present the PG-1 dimer structures in different environments. In the bulk water environment, PG-1 dimers are partially folded β-sheets, while both the antiparallel and parallel β-sheet conformations of the PG-1 dimer are well preserved at the amphipathic interface of the lipid bilayer. Our simulation results provide important guidelines for how the environment supports the β-sheets of the PG-1 dimer and how the dimer activity depends on the β-sheet arrangements. We conducted four simulations starting at different initial configurations, two for antiparallel dimers and two for parallel dimers (page 4). As expected, these simulations do not converge to the same point, since molecules should have conformational and energetic distributions in nature, especially when embedded in the complex membrane environment. Similarly, a recent PG-1 monomer simulation work observed a "kick-shaped" conformation only in one of the two simulations [29]. In the work reported here, while the simulations do not converge to the same conformation, nevertheless the essential features converged: consistently, in both simulations of parallel dimers, the membrane disruption was larger than in the two antiparallel dimer simulations. The parallel β-sheets of the PG-1 dimer induce the membrane disruption effect at the amphipathic interface of the lipid bilayer. Comparing with the recent experimental observation that the parallel PG-1 dimer inserts into POPC lipids [19], our simulations unified experimental observations by revealing that the parallel PG-1 dimer is biologically more active to insert into POPC membrane.

## Methods

A unit cell containing two layers of lipids with almost 65,000 atoms is constructed. The β-sheet of the PG-1 dimer is initially located at the amphipathic region in the top leaflet of the lipid bilayer. Since our simulation method closely follows the previous method of the PG-1 monomer simulation, in this paper we only briefly describe key parameters used for the dimer simulations. The details of the simulation method are described elsewhere [22]. A lipid bilayer containing POPC (palmitoyl-oleyl-phosphatidylcholine) is constructed for the simulations. For the lipid bilayer, 160 POPCs (80 POPCs each side) constitute the lateral cell dimension of 71.3 Å × 71.3 Å. TIP3P waters were added and relaxed through a series of minimization and dynamics. Twelve counter ions (12 *Cl*^-^) were inserted to electrically neutralize the lipid bilayer system, since there are six positively charged amino acid residues in each monomer. To obtain a physiological salt concentration near 100 *mM*, additional 20 *Na*^+ ^and 20 *Cl*^- ^were added.

Our simulation employed the NPAT (constant number of atoms, pressure, surface area, and temperature) ensemble, an effective (time-averaged) surface tension, with a constant normal pressure applied in the direction perpendicular to the membrane. An alternative protocol would involve using variable surface area controlled by constant surface tension with the NPγT (constant number of atoms, pressure, surface tension, and temperature) ensemble, where γ is the applied surface tension. When γ = 0, NPγT is equivalent to NPT. Although experimentally measured macroscopic property of γ should be close to or zero for the non-stressed lipid bilayers [30], a nonzero surface tension must be employed in the NPγT simulations due to the presence of long-wavelength undulation for the microscopic membrane patch [31]. In the NPT ensemble, simulations with CHARMM27 [32] parameter sets reproduce incorrectly reduced surface area [33,34]. However a simulation with a correctly parameterized constant surface area with the NPAT ensemble can be directly comparable to an applied constant surface tension [35,36]. This has led us to use a constant surface area with the NPAT ensemble.

The CHARMM program [32] and Charmm 27 force field were used to construct the set of starting points and to relax the systems to a production-ready stage. In the pre-equilibrium stages, the initial configurations were gradually relaxed, with the peptide held rigid. A series of dynamic cycles were performed with the harmonically restrained peptides, and then the harmonic restraints were gradually diminished with the full Ewald electrostatics calculation and constant temperature (Nosé-Hoover) thermostat/barostat at 310 *K*. The entire pre-equilibration cycle took 5 *ns *to yield the starting point. For the production runs of 20 *ns*, any constraint applied to the peptides was removed, and the simulations were performed with the same parameter sets as used in the pre-equilibrium simulations. The system reached to the equilibration after initial 3–4 *ns*. The NAMD code [37] on a Biowulf cluster at the NIH was used for the starting point with the same Charmm 27 force field in the production simulations. Recent simulation studies suggest that ~10 *ns *duration simulations can reveal details of the interactions of lipid molecules with inner and outer membrane proteins [38,39].

## Authors' contributions

HJ carried out the computational simulations, theoretical calculations, and analysis of the results and drafted the manuscript. BM and RN conceived the study, participated in its design and coordination, and helped to draft the manuscript. All authors read and approved the final manuscript.

## References

[B1] Kokryakov VN, Harwig SS, Panyutich EA, Shevchenko AA, Aleshina GM, Shamova OV, Korneva HA, Lehrer RI (1993). Protegrins: leukocyte antimicrobial peptides that combine features of corticostatic defensins and tachyplesins. FEBS Lett.

[B2] Miyasaki KT, Lehrer RI (1998). Beta-sheet antibiotic peptides as potential dental therapeutics. Int J Antimicrob Agents.

[B3] Fahrner RL, Dieckmann T, Harwig SS, Lehrer RI, Eisenberg D, Feigon J (1996). Solution structure of protegrin-1, a broad-spectrum antimicrobial peptide from porcine leukocytes. Chem Biol.

[B4] Lai JR, Huck BR, Weisblum B, Gellman SH (2002). Design of non-cysteine-containing antimicrobial beta-hairpins: structure-activity relationship studies with linear protegrin-1 analogues. Biochemistry.

[B5] Drin G, Temsamani J (2002). Translocation of protegrin I through phospholipid membranes: role of peptide folding. Biochim Biophys Acta.

[B6] Panchal RG, Smart ML, Bowser DN, Williams DA, Petrou S (2002). Pore-forming proteins and their application in biotechnology. Curr Pharm Biotechnol.

[B7] Sokolov Y, Mirzabekov T, Martin DW, Lehrer RI, Kagan BL (1999). Membrane channel formation by antimicrobial protegrins. Biochim Biophys Acta.

[B8] Gidalevitz D, Ishitsuka Y, Muresan AS, Konovalov O, Waring AJ, Lehrer RI, Lee KY (2003). Interaction of antimicrobial peptide protegrin with biomembranes. Proc Natl Acad Sci USA.

[B9] Sitaram N, Nagaraj R (1999). Interaction of antimicrobial peptides with biological and model membranes: structural and charge requirements for activity. Biochim Biophys Acta.

[B10] Hancock RE, Lehrer R (1998). Cationic peptides: a new source of antibiotics. Trends Biotechnol.

[B11] Chen FY, Lee MT, Huang HW (2003). Evidence for membrane thinning effect as the mechanism for peptide-induced pore formation. Biophys J.

[B12] Zakharov SD, Kotova EA, Antonenko YN, Cramer WA (2004). On the role of lipid in colicin pore formation. Biochim Biophys Acta.

[B13] Mecke A, Lee DK, Ramamoorthy A, Orr BG, Banaszak Holl MM (2005). Membrane thinning due to antimicrobial peptide binding: an atomic force microscopy study of MSI-78 in lipid bilayers. Biophys J.

[B14] Allende D, Simon SA, McIntosh TJ (2005). Melittin-induced bilayer leakage depends on lipid material properties: evidence for toroidal pores. Biophys J.

[B15] Roumestand C, Louis V, Aumelas A, Grassy G, Calas B, Chavanieu A (1998). Oligomerization of protegrin-1 in the presence of DPC micelles. A proton high-resolution NMR study. FEBS Lett.

[B16] Buffy JJ, Waring AJ, Lehrer RI, Hong M (2003). Immobilization and aggregation of the antimicrobial peptide protegrin-1 in lipid bilayers investigated by solid-state NMR. Biochemistry.

[B17] Buffy JJ, Waring AJ, Hong M (2005). Determination of peptide oligomerization in lipid bilayers using 19F spin diffusion NMR. J Am Chem Soc.

[B18] Tang M, Waring AJ, Hong M (2005). Intermolecular packing and alignment in an ordered b-hairpin antimicrobial peptide aggregate from 2D solid-state NMR. J Am Chem Soc.

[B19] Mani R, Tang M, Wu X, Buffy JJ, Waring AJ, Sherman MA, Hong M (2006). Membrane-bound dimer structure of a beta-hairpin antimicrobial peptide from rotational-echo double-resonance solid-state NMR. Biochemistry.

[B20] Mani R, Cady SD, Tang M, Waring AJ, Lehrer RI, Hong M (2006). Membrane-dependent oligomeric structure and pore formation of a beta-hairpin antimicrobial peptide in lipid bilayers from solid-state NMR. Proc Natl Acad Sci USA.

[B21] Harper JD, Lansbury PT (1997). Models of amyloid seeding in Alzheimer's disease and scrapie: mechanistic truths and physiological consequences of the time-dependent solubility of amyloid proteins. Annu Rev Biochem.

[B22] Jang H, MA B, Woolf TB, Nussinov R (2006). Interaction of protegrin-1 (PG-1) with lipid bilayers: membrane thinning effect. Biophys J.

[B23] Axelsen PH, Kaufman BK, McElhaney RN, Lewis RN (1995). The infrared dichroism of transmembrane helical polypeptides. Biophys J.

[B24] Han X, Tamm LK (2000). A host-guest system to study structure-function relationships of membrane fusion peptides. Proc Natl Acad Sci USA.

[B25] Berneche S, Nina M, Roux B (1998). Molecular dynamics simulation of melittin in a dimyristoylphosphatidylcholine bilayer membrane. Biophys J.

[B26] Lague P, Roux B, Pastor RW (2005). Molecular dynamics simulations of the influenza hemagglutinin fusion peptide in micelles and bilayers: conformational analysis of peptide and lipids. J Mol Biol.

[B27] Buffy JJ, Hong T, Yamaguchi S, Waring AJ, Lehrer RI, Hong M (2003). Solid-state NMR investigation of the depth of insertion of protegrin-1 in lipid bilayers using paramagnetic Mn2+. Biophys J.

[B28] Yamaguchi S, Hong T, Waring A, Lehrer RI, Hong M (2002). Solid-state NMR investigations of peptide-lipid interaction and orientation of a beta-sheet antimicrobial peptide, protegrin. Biochemistry.

[B29] Khandelia H, Kaznessis YN (2007). Structure of the antimicrobial beta-hairpin peptide protegrin-1 in a DLPC lipid bilayer investigated by molecular dynamics simulation. Biochim Biophys Acta.

[B30] Jahnig F (1996). What is the surface tension of a lipid bilayer membrane?. Biophys J.

[B31] Feller SE, Pastor RW (1996). On simulating lipid bilayers with an applied surface tension: periodic boundary conditions and undulations. Biophys J.

[B32] Brooks BR, Bruccoleri RE, Olafson BD, States DJ, Swaminathan S, Karplus M (1983). Charmm - a program for macromolecular energy, minimization, and dynamics calculations. J Comp Chem.

[B33] Jensen MO, Mouritsen OG, Peters GH (2004). Simulations of a membrane-anchored peptide: structure, dynamics, and influence on bilayer properties. Biophys J.

[B34] Benz RW, Castro-Roman F, Tobias DJ, White SH (2005). Experimental validation of molecular dynamics simulations of lipid bilayers: a new approach. Biophys J.

[B35] Feller SE, Pastor RW (1999). Constant surface tension simulations of lipid bilayers: The sensitivity of surface areas and compressibilities. J Chem Phys.

[B36] Skibinsky A, Venable RM, Pastor RW (2005). A molecular dynamics study of the response of lipid bilayers and monolayers to trehalose. Biophys J.

[B37] Phillips JC, Braun R, Wang W, Gumbart J, Tajkhorshid E, Villa E, Chipot C, Skeel RD, Kale L, Schulten K (2005). Scalable molecular dynamics with NAMD. J Comp Chem.

[B38] Deol SS, Bond PJ, Domene C, Sansom MS (2004). Lipid-protein interactions of integral membrane proteins: a comparative simulation study. Biophys J.

[B39] Domence C, Bond PJ, Deol SS, Sansom MSP (2003). Lipid/Protein Interactions and the Membrane/Water Interfacial Region. J Am Chem Soc.

